# How to Develop Renewable Power in China? A Cost-Effective Perspective

**DOI:** 10.1155/2014/946932

**Published:** 2014-01-21

**Authors:** Rong-Gang Cong, Shaochuan Shen

**Affiliations:** ^1^School of Economics and Management, North China Electric Power University, Beijing 102206, China; ^2^Centre for Environmental and Climate Research (CEC), Lund University, 22362 Lund, Sweden; ^3^State Key Laboratory Breeding Base of Green Chemistry Synthesis Technology, College of Chemical Engineering and Materials Science, Zhejiang University of Technology, Hangzhou 310014, China

## Abstract

To address the problems of climate change and energy security, Chinese government strived to develop renewable power as an important alternative of conventional electricity. In this paper, the learning curve model is employed to describe the decreasing unit investment cost due to accumulated installed capacity; the technology diffusion model is used to analyze the potential of renewable power. Combined with the investment cost, the technology potential, and scenario analysis of China social development in the future, we develop the Renewable Power Optimization Model (RPOM) to analyze the optimal development paths of three sources of renewable power from 2009 to 2020 in a cost-effective way. Results show that (1) the optimal accumulated installed capacities of wind power, solar power, and biomass power will reach 169000, 20000, and 30000 MW in 2020; (2) the developments of renewable power show the intermittent feature; (3) the unit investment costs of wind power, solar power, and biomass power will be 4500, 11500, and 5700 Yuan/KW in 2020; (4) the discounting effect dominates the learning curve effect for solar and biomass powers; (5) the rise of on-grid ratio of renewable power will first promote the development of wind power and then solar power and biomass power.

## 1. Introduction

**Figure 1:**
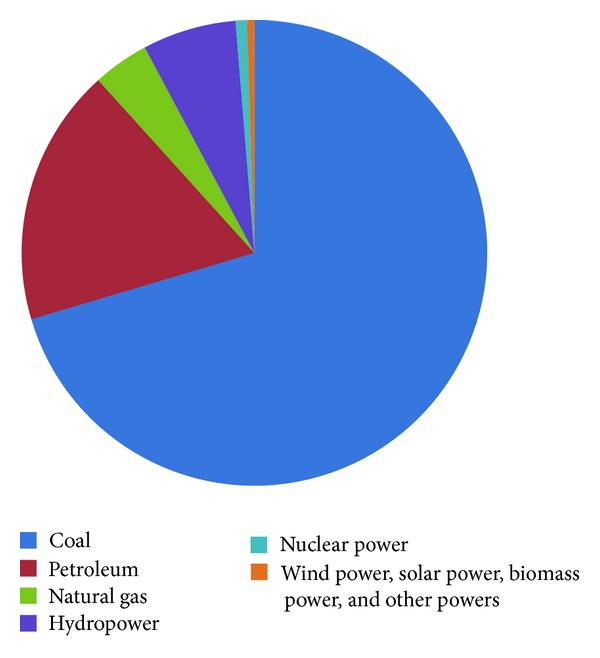
China energy consumption in 2009. Source: China energy statistical yearbook 2010.

According to the twelfth five-year plan about renewable energy development published by State Council of China in 2012, the proportion of nonfossil energy in primary energy consumption will be increased to 11.4% in 2015 and 15% in 2020 [1]. However, China renewable energy consumption in 2009 was only 7% of primary energy consumption (Figure 1). Furthermore, because hydropower which occupied 92% of renewable energy (6.5% of primary energy consumption) gets saturated, the development of renewable power (mainly wind power, solar power, and biomass power in this paper) faces big challenges.

The development of renewable power is also good for the sustainability of energy supply [2] but subject to a number of factors including economic factors (e.g., development and utilization cost [3]), technological factors (e.g., maturity of technology [4]), and other factors (e.g., resource endowment that is the natural resources within the borders of a country [5], social acceptance (i.e., the development of wind energy has the visual impact on landscapes which is facing vivid debates on local and sometimes national levels) [6], environmental constraints [7], etc.). Particularly, in countries with characteristics of planned economy, such as China, the development of renewable power must meet the plan for national economy which is different from market-economy countries, such as USA.

Because the construction of grid is a long-term project, scientific analysis and forecasting of the size and structure of the renewable power are important for the planning of grid construction in advance, which will reduce the possibility of overinvestment or underinvestment. Therefore, this paper has profound practical background and high application value.

This paper tries to analyze the optimal development path of renewable power in a cost-effective way; that is, (1) the development of renewable power is subject to economic affordability (i.e., the investment in each year should be less than a certain proportion of GDP), while the unit investment cost has the decreasing trend due to the increase of accumulated installed capacities (the learning curve effect); (2) the development of renewable power is constrained by relevant technology maturity (the technology diffusion effect); (3) the development of renewable power should meet the requirement of the plan for national economy. Considering the factors above, we construct the Renewable Power Optimization Model (RPOM) to analyze the optimal development paths of three main sources of renewable power (wind power, solar power, and biomass power) in China in a cost-effective way.

The paper is structured as follows. We begin with a literature review at Section 2, followed by the descriptions of modeling about the cost and technology maturity of renewable power in Section 3.  Section 4 describes the Renewable Power Optimization Model (RPOM) which utilizes the results from Section 3. In Section 5, we present the main results from the model and do a sensitivity analysis for main uncertainties. Finally, the main conclusion and possible future work are presented.

## 2. Literature Review

The economic literature relevant to development of renewable power can be classified into two categories: (1) research on how the potential factors could affect the development of renewable power between each other, that is, economic cost, technology maturity, and so forth; (2) the comprehensive planning model of renewable power taking the different impacting factors mentioned above into account.

### 2.1. Impacting Factors of Development of Renewable Power

There are a lot of potential factors which affect the development of renewable power. Among them the economic factor and the technology factor are especially important which attract widespread attention.

The economic cost (e.g., unit investment cost) is a main barrier for scale development of renewable power [8–10]. Most renewable power technologies are relatively new and have not yet become fullycommercialized. This problem is especially acute in China because the government wants to keep the price of power low to support economic development [11]. According to Cong and Wei's study [12], unit generation cost of coal-fired power was 350 Yuan/KKWH, while unit generation costs of wind power and solar power were 620 and 1900 Yuan/KKWH separately (1 Chinese Yuan is equal to 0.16 US Dollar roughly in 2013). The scale development of renewable power faces a strong market competition of conventional power.

However, during the past few decades, the cost of renewable power has had a substantial decline [13]. A relatively mature method to forecast the economic cost of renewable power is the learning curve method [14, 15]. The core idea of the learning curve is that unit costs decrease with increasing experience (e.g., accumulated production). Neij used the learning curve method to analyze the prospects of costs of wind turbines and photovoltaic modules and found that renewable power has larger potential to reduce costs than conventional power [16]. Goldemberg applied the learning curve to analyze the cost of bioenergy and found that the cost of ethanol declined an average 6% per year when its production increased from 0.9 billion gallons to 4.2 billion gallons [17]. Although there is still a great debate about the parameters and the specific forms of function, the learning curve is still a mainstream method among other methods (e.g., engineering assessment [18]) and can provide more robust cost projections when relevant data is available.

Another main barrier for scale development of renewable power is relevant technology support [19, 20]. This problem is more serious in developing countries, such as China and India, because most technologies of renewable power in these countries are still in development [21]. One important indicator to describe technology support is the degree of technology maturity which depends on its stage on the life cycle. Generally, the life cycle of renewable power technology includes the four stages: infancy, growth, maturity, and recession [22]. The model for explaining the maturity degree of new technologies over time is often referred to as the technology diffusion model. A typical technology diffusion model is the S-curve model [23]. This is because usually renewable power technologies' diffusion first takes up slowly, then rises rapidly, and finally increases slowly to satiation. There are several commonly suggested S-curve models, such as the Bass model [24], the Gompertz model [25], the Logistic model [26], and the Pearl model [27].

### 2.2. Comprehensive Utilization of Renewable Energy Generation

Because the development of renewable power is subject to a lot of factors, its comprehensive utilization must balance among these factors [28]. Jain [29] pointed out that from the perspectives of energy production and utilization the concept of Integrated Renewable Energy Systems (IRES), which utilize different sources of energy, such as wind, and solar heat, to satisfy various energy needs [30], is feasible. Ramakumar et al. [31] developed the IRES model using linear programming methods whose objective was to minimize the annual cost under the constraints of resource.

Ashenayi and Ramakumar [30] designed the IRES model based on the loss of power-supply probability (LPSP) as the key system parameter and minimization of the initial capital investment. Iniyan and Jagadeesan [32] developed an Optimal Renewable Energy Model (OREM) taking the constraints of social acceptance, resource limitation, demand, and reliability factors into account. They give the allocation of renewable energy sources for lighting, cooking, pumping, heating, cooling, and transportation end-uses for the years 2020-2021 in India. Iniyan et al. [33] studied the optimal utilization of renewable energy in India based on modified econometric model, mathematical programming model, and OREM model. It fully considered the impact of cost, efficiency, social acceptance, reliability, potential, and environment.

In this paper we conduct an integrated planning for renewable power based on a mathematical optimization [34, 35] inspired by the idea of IRES (Section 2.2). The proposed model explicitly considers the impacts of learning curve and technology maturity of renewable power technologies (Section 2.1) on their scale developments. The model is also dynamic to optimize the development path of renewable power over periods.

## 3. Cost and Technology Maturity of Renewable Power

The learning curve models and technology diffusion models for power from three renewable sources (wind power, solar power, and biomass power) are developed in Cong's study [36]. The data used is obtained from different sources including Chinese Wind Energy Association and World Wind Energy Council. The time span is from 1996 to 2008. The fundamental of the learning curve model that we use is to describe the relationship between cumulative installed capacity and unit investment cost. Based on the learning curve model, we can forecast the unit investment cost of renewable power given their cumulative installed capacity in the future. The learning rates are calculated according to historical data. The technology diffusion model can consider the resource potential and give out the maximum possible cumulative installed capacity (potential) in any period based on the historical data. The learning curve model and the technology diffusion model have been validated to reflect the historical data well.

## 4. Renewable Power Optimization Model (RPOM)

The model is used to forecast the size and structure of China renewable power (mainly wind power, solar power, and biomass power) in 2015 and 2020 in a cost-effective way. The model is inspired by IRES and OREM models and basically a dynamic optimization model. The objective function is to minimize the total investment cost for installed capacities of renewable power (which is different from the objective of optimization model developed by Cong [36]) given a set of constraints: (1) the installed capacity of each renewable power is less than its potential at that time (given by the technical maturity model); (2) the cost of installed capacity of renewable power is affordable for the total economy in each year; (3) the total size of renewable power is less than a certain proportion of total generation (grid limit); (4) each renewable power must meet the national plans over years. Decision variables are the added capacity of renewable power from 2009 to 2020. The specific model is as follows:

(1)  max⁡   ∑t=20092020rt−2008×(Aw(t)×Cw(t)+As(t)               ×Cs(t)+Ab(t)×Cb(t)) subject  to Nw(t)≤Mw(t),      Ns(t)≤Ms(t),      Nb(t)≤Nb(t),      Aw(t)×Cw(t)+As(t)×Cs(t),        +Ab(t)×Cb(t)≤γ×G(t),      Nw(t)+Ns(t)+Nb(t)≤μI(t),      Nw(2010)≥Pw(2010),      Ns(2010)≥Ps(2010),      Nb(2010)≥Pb(2010)      Nw(2020)≥Pw(2020),      Ns(2020)≥Ps(2020),      Nb(2020)≥Pb(2020),      Nw(t)=Nw(t−1)+Aw(t),      Ns(t)=Ns(t−1)+As(t),      Nb(t)=Nb(t−1)+Ab(t),      Cw(t)=Cw,min⁡+Cw(t0)Nw(t−t0)ϕw,      Cs(t)=Cs,min⁡+Cs(t0)Ns(t−t0)ϕs,      Cb(t)=Cb,min⁡+Cb(t0)Nb(t−t0)ϕb,      Aw(t)≥0,       As(t)≥0,       Ab(t)≥0,

where *r* is the discounting rate; *M*_*w*_ is the potential (maximum possible accumulated installed capacity) of wind power; *M*_*s*_ is the potential of solar power; *M*_*b*_ is the potential of biomass power; *A*_*w*_ is the added installed capacity of wind power; *A*_*s*_ is the added installed capacity of solar power; *A*_*b*_ is the added installed capacity of biomass power; *γ* is the proportion of GDP that can be used for investment in renewable power; *G*(*t*) is the GDP in year *t*; *μ* is the maximum on-grid proportion of renewable power; *I*(*t*) is the total installed capacity in year *t*; *P*_*w*_ is the planned installed capacity of wind power; *P*_*s*_ is the planned installed capacity of solar power; *P*_*b*_ is the planned installed capacity of biomass power; *C*_*w*,min⁡_ is the minimum unit cost for installed capacity of wind power; *C*_*s*,min⁡_ is the minimum unit cost for installed capacity of solar power; *C*_*b*,min⁡_ is the minimum unit cost for installed capacity of biomass power; *C*_*w*_(*t*) is the unit investment cost for wind power in year *t* (10^4^ Yuan/KW); *C*_*s*_(*t*) is the unit investment cost for solar power in year *t* (Yuan/MW); *C*_*b*_(*t*) is the unit investment cost for biomass power in year *t* (10^4^ Yuan/KW); *N*_*w*_(*t*) is the cumulative installed capacity of wind power in the year *t*; *N*_*s*_(*t*) is the cumulative installed capacity of solar power in year *t*; *N*_*b*_(*t*) is the cumulative installed capacity of biomass power in the year *t*. *A*_*w*_(*t*), *A*_*s*_(*t*), and *A*_*b*_(*t*) are decision variables.

## 5. Model Results and Sensitivity Analysis

In this section, first we provide the main results of the model including the accumulated installed capacities, the added installed capacities, and the unit investment costs of power from three renewable sources. Second, we do the sensitivity analysis for the main uncertainties: the discounting rate and the on-grid ratio.

### 5.1. The Model Results

**Figure 2:**
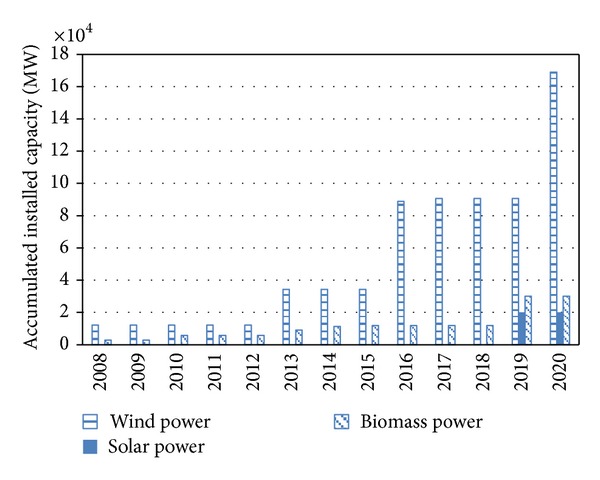
The accumulated installed capacities of three renewable sources of power.

The accumulated installed capacities of wind power, solar power, and biomass power will reach 34294, 145, and 11887 MW in 2015, and reach 169000, 20000, and 30000 MW in 2020 (Figure 2). According to the draft of “new energy promotion plan” and “renewable energy long-term development plan”, the expected installed capacity of wind power, solar power, and biomass power will reach 150000, 20000, and 30000 MW in 2020. The optimal installed capacity of wind power is higher than the planning scenario by 12.7%, which means that, compared to the solar power and biomass power, wind power has larger growth potential.

**Figure 3:**
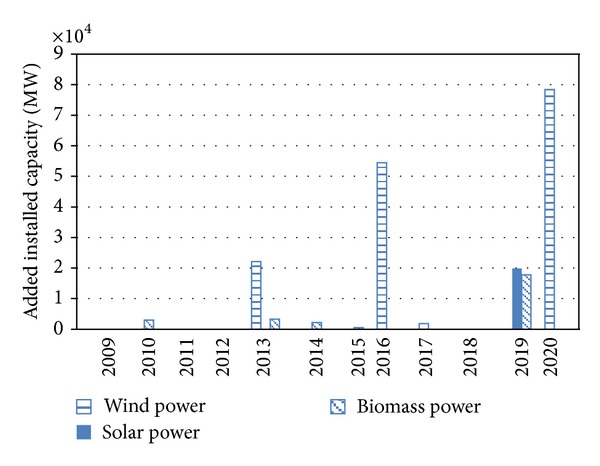
The added installed capacities of three renewable sources of power.

From the cost-effective perspective, the growths of renewable power show the intermittent feature: wind power has large increase in 2013, 2016, and 2020; solar power and biomass power have large increase in 2019 (Figure 3). This is mainly because the investments must be made in some years to satisfy the planned targets. However, the investments are not made before the specific years to save money considering the time value of money. Actually there are two effects when doing the investment decisions: (1) the learning curve effect that early investment will decrease the cost afterwards and (2) the discounting effect that the late investment will save the interest. In this case, we find that the discounting effect dominates the learning curve effect for solar power and biomass power.

**Figure 4:**
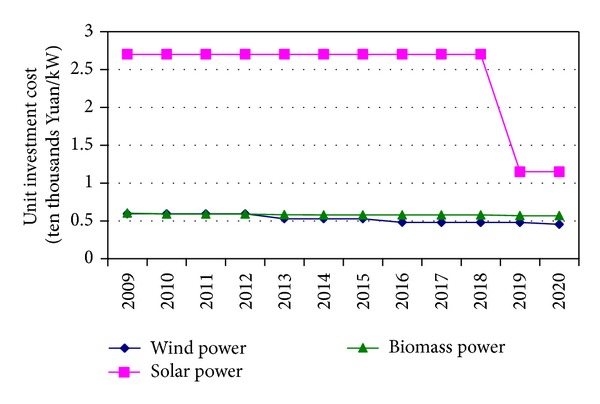
The unit investment costs of power from three renewable sources.

The unit investment cost of solar power is the highest in the three renewable sources of power. Because the investment for solar power is small in the initial periods, its unit investment cost shows slow decline until 2018. The unit investment cost of wind power is slightly lower than biomass power over the periods. The unit investment costs of wind power, solar power and biomass power will be 0.53, 2.7, and 0.58 ten thousand Yuan/KW in 2015, and 0.45, 1.15, and 0.57 ten thousand Yuan/KW in 2020 (Figure 4).

### 5.2. Sensitivity Analysis of the Main Uncertainties

The optimal sizes of renewable power are determined by a lot of factors, including the impact of technology diffusion, unit investment cost, power grid technology, macro investment ratio, and discounting rate. And there are great uncertainties in these factors. To obtain robust results, we use the sensitivity analysis methods for two main uncertainties: discounting rate and on-grid ratio of renewable power. We do not do the sensitivity analysis for the learning curve model and diffusion model because their parameters are mainly obtained according to historical data.

**Figure 5:**
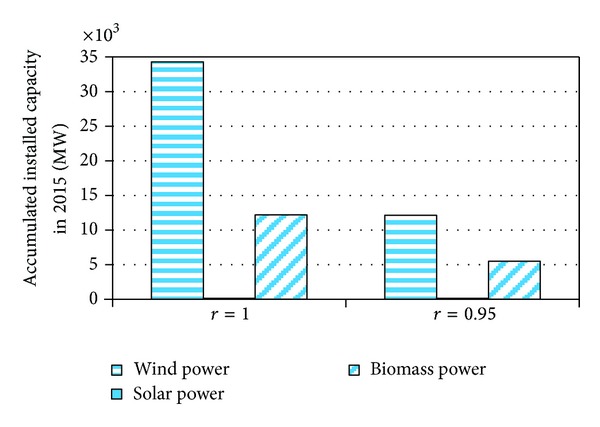
The impact of discounting rate, *r*, on the accumulated installed capacities of renewable power in 2015.

When the discounting rate decreases from 1 (which we use in the model above) to 0.95, the total installed capacities of renewable power will become smaller (Figure 5), which means that the investment for renewable power will lag when considering the time value of money.

**Figure 6:**
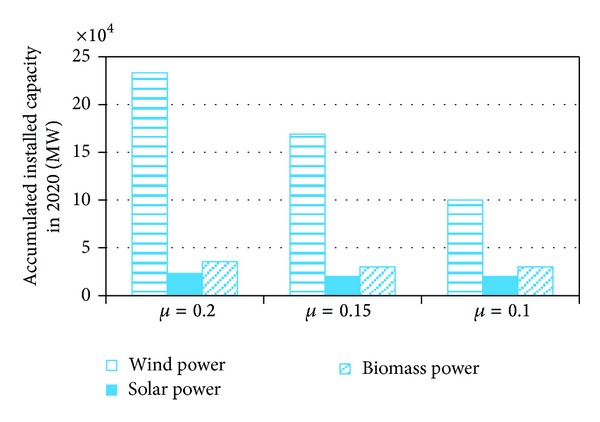
The impact of *μ* on the accumulated installed capacities of renewable power in 2020.

When the on-grid ratio of renewable power is 0.1, the planned installed capacity can fully achieve the target. When the on-grid ratio of renewable power is 0.15 (which we use in the model above), the potential of wind power should be utilized; the installed capacity of wind power in 2020 increases from 100000 MW to 169000 MW. However, if the on-grid ratio of renewable power rises to 0.2 further, not only the potential of wind power but also the potential of solar power and biomass power should be exploited; the accumulated installed capacities of wind power, solar power, and biomass power in 2020 will be 233321, 23173, and 35506 MW (Figure 6).

## 6. Conclusions

Considering the economic cost, technology maturity of renewable energy generation, Chinese social development, and other relevant factors, we construct the optimal development model of Chinese renewable power and obtain the solutions. The main conclusions are as follows.

1. The accumulated installed capacities of wind power, solar power, and biomass power will be 34294, 145 and 12195 MW in 2015 and 169000, 20000, and 30000 MW in 2020. Compared with relevant plans, it is found that wind power has larger development potential than solar power and biomass power.
2. The optimal added installed capacities show the intermittent feature: wind power has a relatively large increase in 2013, 2016, and 2020; solar power and biomass power have a relatively large increase in 2019. It is found that the discounting effect dominates the learning curve effect for solar power and biomass power.
3. The unit investment cost of solar power is the highest among the three renewable sources of generation. Because the investment for solar power is small in the initial periods, its unit investment cost shows slow decline until 2018. The unit investment cost of wind power is slightly lower than biomass power over the periods. The unit investment costs of wind power, solar power and biomass power are 5300, 27000, and 5800 Yuan/KW in 2015 and 4500, 11500, and 5700 Yuan/KW in 2020. There is a large potential for solar power to decrease its unit investment cost in 2020 due to the learning curve effect.
4. If the time value of money is considered, the investment of renewable power will lag. When the on-grid proportion of renewable power is 0.1, the current plan can fully achieve the target; when the on-grid proportion of renewable power is 0.15, the development potential of wind power should be exploited; when the on-grid proportion of renewable power rises to 0.2, not only the potential of wind power but also the potential of solar power and biomass power should be exploited.

The dynamic optimization model used in this paper considers the relationships among internal factors of development of renewable power. For example, installed capacity and cost reduction interact between each other, which is so-called the learning curve effect. And the time value of money (the discounting effect) also complicates the decision. This paper also contributes to the current literature because it studies how to satisfy the targets presented by the planned economy (such as China), which is absolutely different from market economy.

However, this research also has some limitations which need to be studied in the future. For example, the learning curve model does not consider the capacities of renewable power produced for exports. The possible correlation between the learning curves and the technology diffusion is not taken into account either.
